# Rethinking immunotherapy sequencing in multiple myeloma: a conceptual adaptive immunotherapy sequencing model

**DOI:** 10.3389/fimmu.2026.1897153

**Published:** 2026-10-15

**Authors:** Aliaa Khalili

**Affiliations:** Department of Hemato-Oncology, Al-Quds University School of Medicine, Jerusalem, Palestine

**Keywords:** adaptive immunotherapy sequencing model, antigen escape, bispecific antibodies, CAR-T cell therapy, multiple myeloma, relapsed/refractory, T-cell fitness

## Abstract

**Background:**

Chimeric antigen receptor T-cell (CAR-T) therapy and bispecific antibodies (BsAbs) have transformed treatment for relapsed/refractory multiple myeloma (RRMM). Contemporary guidelines increasingly incorporate prior antigen-directed exposure, disease kinetics, patient fitness, toxicity, access, and patient preference. However, a prospectively validated framework that jointly operationalizes immune fitness, tumor antigen status, and treatment feasibility for sequencing T-cell–redirecting therapies is not available.

**Main body:**

This Perspective introduces the Adaptive Immunotherapy Sequencing Model (AISM), an author-proposed framework for research and multidisciplinary discussion. AISM proposes that sequencing should be studied in relation to two evolving biological domains: the capacity of the host T-cell compartment to support therapy and the probability that the relevant tumor antigen remains therapeutically actionable. The model uses provisional 0–10 research constructs for T-cell fitness and antigen-escape risk to make these domains explicit. AISM hypothesizes that, for eligible patients with early RRMM and feasible access, referral for BCMA-directed CAR-T before prolonged exposure to T-cell–redirecting BsAbs may preserve a potentially favorable treatment option. It also recognizes that off-the-shelf BsAbs may be necessary when disease tempo, frailty, access, or patient preference make CAR-T impracticable. After prior BCMA-directed therapy, an antigen-switching approach is biologically plausible, but antigen loss is not universal and other mechanisms of resistance must be considered.

**Conclusions:**

AISM provides a testable framework for prospective comparative studies. Such studies should evaluate whether biologically informed sequencing improves progression-free survival (PFS), while also assessing overall survival, toxicity, infections, treatment-free time, feasibility, and patient-reported outcomes.

## 1 Introduction: defining the unmet sequencing problem

T-cell–redirecting therapies have changed the therapeutic landscape of RRMM. BCMA-directed CAR-T products and BsAbs, as well as BsAbs directed at G protein–coupled receptor, class C, group 5, member D (GPRC5D), can produce deep responses in heavily pretreated disease (1–5). Their increasing availability in earlier relapse has shifted the clinical question from whether to use T-cell redirection to how, when, and in what order to use it.

Current guidelines and expert recommendations do not treat these decisions as wholly interchangeable. They increasingly consider previous exposure to BCMA- or GPRC5D-directed treatment, rate of disease progression, treatment availability, fitness, toxicity, and patient preferences (6). Nevertheless, these factors are usually considered separately, and no prospectively validated tool integrates host immune competence, tumor antigen status, and practical feasibility to guide sequencing. This is the specific gap addressed by AISM.

The Adaptive Immunotherapy Sequencing Model (AISM) is proposed by the author in this Perspective as a conceptual framework for research and multidisciplinary discussion. It is intended to make biological assumptions transparent, generate testable sequencing hypotheses, and identify the measurements that would be needed to validate them. Treatment selection must remain individualized and incorporate regulatory indication, eligibility for lymphodepletion and cell therapy, comorbidity and frailty, disease tempo and tumor burden, prior treatments, anticipated toxicity, local capacity, cost and reimbursement, and the informed preferences of the patient.

AISM rests on two linked hypotheses. First, the quality and availability of T cells may influence the activity of both CAR-T therapy and BsAbs, although their dependence on the patient’s native T-cell compartment differs. Second, antigen-directed treatment exerts selective pressure, so the value of reusing a target may depend on tumor antigen state and the mechanism of prior treatment failure. Both hypotheses have biological and emerging clinical support, but neither establishes that an AISM-directed sequence improves patient outcomes. The purpose of this article is therefore to define the model, its boundaries, and a prospective path to test it.

## 2 Biological and clinical considerations in sequencing T-cell–redirecting therapy

### 2.1 CAR-T therapy and BsAbs are related but not biologically interchangeable

CAR-T therapy and BsAbs both redirect cytotoxic T cells toward myeloma, but they do so through distinct therapeutic architectures. CAR-T therapy requires leukapheresis, manufacturing, bridging therapy when necessary, lymphodepletion, and a single infusion of an ex vivo engineered cellular product. Its effectiveness can be influenced by the phenotype and functional reserve of cells collected at apheresis, product attributes, *in vivo* expansion and persistence, tumor burden, and the marrow or extramedullary microenvironment. In exchange for this complex pathway, CAR-T can provide a treatment-free interval after the acute treatment phase.

BsAbs are off-the-shelf agents that repeatedly engage endogenous CD3-positive T cells with a myeloma antigen. They permit rapid treatment initiation and avoid manufacturing delay, but conventional schedules may involve sustained exposure and recurrent clinic visits. Their efficacy is influenced by the pretreatment T-cell landscape, effector-to-target balance, antigen state, dose exposure, and tumor microenvironment. In MajesTEC-1 correlative analyses, features of immune fitness were associated with response to teclistamab, supporting the clinical relevance of host immune context (7, 8). These differences mean that the sequencing question is not simply “CAR-T versus BsAb”; it also concerns urgency, capacity to complete the CAR-T pathway, duration of immune pressure, and the biological consequences of prior exposure.

### 2.2 T-cell fitness is multidimensional

In this Perspective, T-cell fitness denotes the capacity of a patient’s T-cell compartment to expand, persist, traffic, and exert cytotoxic function after therapeutic redirection. It is not a single laboratory value. Clinical and translational evidence supports associations between T-cell state and response, including the composition of pre-existing T-cell populations, activation/exhaustion programs, and immune reserve (7–9). Prior therapy may contribute through cumulative lymphotoxic exposure, but it is only one determinant.

Additional factors can influence T-cell fitness or the apparent activity of T-cell–redirecting treatment. These include age and frailty; tumor burden and disease kinetics; inflammatory state; recent or prolonged corticosteroid exposure; cytopenias; prior transplantation; the frequency and phenotype of regulatory, exhausted, or senescent T-cell populations; and suppressive features of the tumor microenvironment (7, 9, 10). Some are readily measured in practice, whereas others remain research measures. The independent predictive contribution of these factors, and their interactions, have not been prospectively calibrated for CAR-T or BsAb sequencing. AISM therefore treats them as candidate domains rather than as established determinants of treatment choice.

### 2.3 Antigen escape is important, but it is not the sole explanation for resistance

Antigen loss, downregulation, epitope alteration, and increased shedding of soluble BCMA (sBCMA) are biologically credible mechanisms of resistance to BCMA-directed therapy (11–14). However, complete BCMA loss appears uncommon after some CAR-T exposures, and detectable surface antigen does not guarantee response (13, 14). Resistance may instead reflect inadequate CAR-T expansion or persistence, T-cell dysfunction, a low effector-to-target ratio, immunosuppressive tumor microenvironment, tumor-intrinsic anti-apoptotic programs, spatial heterogeneity, or an antigen alteration that is not captured by a standard surface-expression assay (11, 13, 14). Similar principles may apply to GPRC5D-directed therapy.

Accordingly, a previous BCMA-directed treatment should not be equated automatically with BCMA-negative disease, and target switching should not be presented as mandatory in every case. Rather, AISM treats prior target exposure as a reason to assess the mechanism and context of resistance, consider an alternative target, and avoid assuming that immediate recycling of the same target will be equally effective. The European Myeloma Network (EMN) similarly notes that sequential use of agents against the same antigen can be feasible, while target switching has generally shown greater activity in available studies (6).

### 2.4 Treatment intensity, infection, and durability must be balanced

Continuous exposure to a BsAb can sustain disease control but may be associated with T-cell dysfunction, hypogammaglobulinemia, cytopenias, and infection risk (15–20). These risks require active prevention and monitoring rather than an assumption that continuous therapy is intrinsically inappropriate. Dose modification, interval extension, immunoglobulin replacement, antimicrobial prophylaxis, and treatment interruption are being studied or used in selected contexts (18–20). Evidence for fixed-duration BsAb therapy is still limited and should not be treated as an established standard.

Conversely, the potential treatment-free interval after CAR-T should be balanced against relapse, prolonged cytopenias, infections, cytokine-release syndrome, immune effector cell–associated neurotoxicity, and delayed adverse events. CAR-T selection therefore requires careful assessment of acute and late risks, not merely a preference for treatment-free time (1, 2, 19). AISM considers treatment-free time a patient-relevant outcome, not proof of superior long-term benefit.

## 3 The adaptive immunotherapy sequencing model

AISM is a two-domain, hypothesis-generating framework. It asks clinicians and investigators to consider (1) whether the host T-cell compartment is likely to support the proposed mode of T-cell redirection and (2) whether the selected target remains actionable. A third, non-biological layer—clinical feasibility—modifies any biologically preferred option. The framework does not replace standard eligibility assessment, current guidelines, or multidisciplinary review. **Figure 1** summarizes this proposed structure and its requirement for prospective validation.

**Figure 1:**
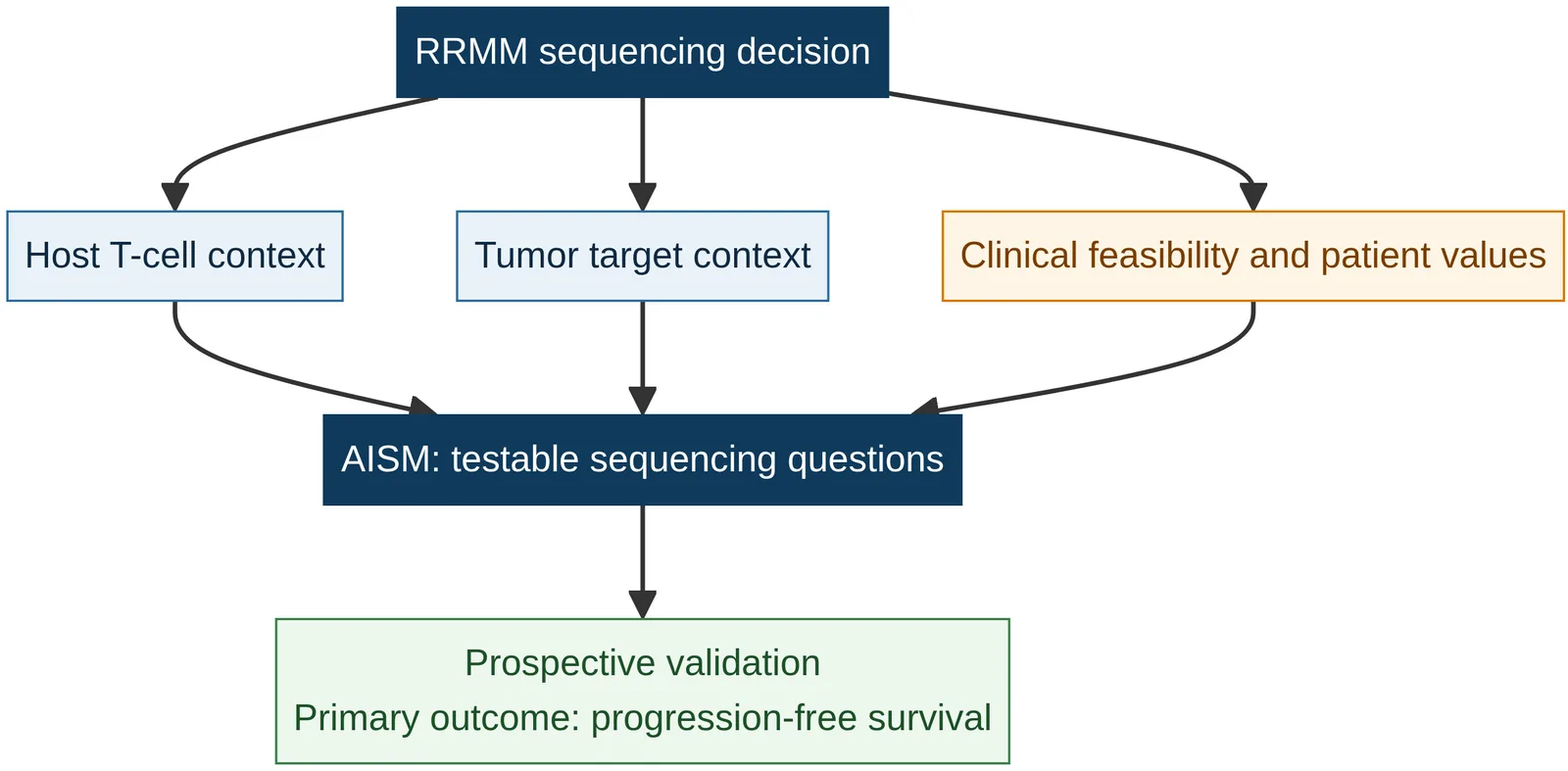
Conceptual AISM framework. At a sequencing decision, the model considers host T-cell context, tumor target context, and clinical feasibility and patient values. These inputs generate testable sequencing questions for prospective validation; the framework is hypothesis-generating and does not define a treatment threshold.

The model’s proposed 0–10 constructs are deliberately provisional. They were included to make the hypothesized domains operationalizable for future study, not to claim that their components have validated weights. Their relative contributions have not been estimated in a derivation cohort. The numbers are transparent ordinal placeholders, and an illustrative boundary of 7 is a research calibration target, not a treatment threshold. These constructs require prospective derivation and external validation before routine clinical use.

### 3.1 Provisional T-cell fitness construct

The provisional T-cell fitness construct is anchored at 0 (least favorable immune context) and 10 (most favorable immune context). The suggested maximum contributions are 4 points for prior immune-impacting treatment exposure, 3 points for disease/inflammatory burden, and 3 points for immune reserve. The greater 4-point allocation for prior exposure does not assert that it is more important than the other domains. It is a pragmatic, hypothesis-level choice because longitudinal treatment history is uniformly available and may summarize cumulative immune perturbation; it requires empirical testing against direct immune phenotyping and clinical outcome. Disease burden and immune reserve receive equal 3-point allocations because both may affect the immediate effector-to-target context and are partly measurable at the treatment decision point. The candidate domains, proposed contributions, and measurements for T-cell fitness are summarized in **Table 1**.

**Table 1:** Provisional T-cell fitness construct.

Candidate domain	Proposed contribution	Candidate measurements	Rationale and evidence boundary
Prior immune-impacting exposure	0–4	Number, recency, and duration of alkylator-, transplant-, corticosteroid-, and prior T-cell–redirecting therapy exposures	Captures cumulative treatment history; not a validated measure of T-cell function (9, 10).
Disease and inflammatory burden	0–3	Disease kinetics, marrow involvement, circulating plasma cells, LDH, bulky plasmacytoma, extramedullary disease, inflammatory markers	High burden can affect clinical urgency and effector-to-target balance; individual measures require prospective calibration.
Immune reserve	0–3	Absolute lymphocyte count, lymphocyte subsets, CD4/CD8 ratio, selected memory/exhaustion markers where available	Immune phenotype is associated with activity of T-cell engagers, but no standardized clinical panel or universal cutoff exists (7–9).

The proposed contributions are ordinal research scaffolds for prospective derivation and calibration. They are not validated weights, cutoffs, or treatment thresholds; frailty, organ function, access, and patient goals are considered clinical modifiers.

Frailty is intentionally not embedded within this construct. Frailty, performance status, organ function, social support, and patient goals are clinical modifiers that can determine suitability for lymphodepletion, hospitalization, or continuous therapy even when laboratory immune features appear favorable. The illustrative boundary of 7 is intended for prospective evaluation, rather than as a binary treatment rule.

### 3.2 Provisional antigen-escape construct and practical measurement limits

The provisional antigen-escape construct is anchored at 0 (low concern that the target is non-actionable) and 10 (high concern). It assigns up to 5 points to the nature, duration, recency, and outcome of prior BCMA-directed therapy; up to 3 points to tumor antigen findings; and up to 2 points to sBCMA. The 5-point allocation for prior exposure reflects the clinical availability of that information and its role as a proxy for selective pressure. It is not evidence that prior exposure outweighs antigen measurement or sBCMA biologically. The allocation is intended to be replaced by model-derived coefficients in a prospective cohort. The candidate domains, proposed contributions, and measurements for antigen escape are summarized in **Table 2**.

**Table 2:** Provisional antigen-escape construct.

Candidate domain	Proposed contribution	Candidate measurements	Rationale and evidence boundary
Prior BCMA-directed exposure	0–5	Modality, duration, interval since therapy, depth and duration of response, and pattern of relapse	Readily available proxy for prior selective pressure; does not prove antigen loss (6, 11, 13).
Tumor antigen findings	0–3	BCMA expression on CD138-positive plasma cells by validated flow cytometry; tissue assessment for accessible extramedullary relapse; molecular testing where available	May identify loss or altered expression, but surface expression alone is not a complete resistance assay (12–14).
Soluble BCMA	0–2	sBCMA concentration and, where validated, ratio to membrane BCMA	Can affect some BCMA-targeting T-cell engagers and has shown association with refractoriness; assay and treatment-specific interpretation remain unsettled (14).

The proposed contributions are ordinal research scaffolds for prospective derivation and calibration. They are not validated weights, cutoffs, or treatment thresholds; clinical feasibility and patient values remain essential modifiers.

Measurement of surface antigen density by flow cytometry is feasible in experienced laboratories but is not standardized as a routine sequencing test. It depends on obtaining a representative marrow or tissue sample, plasma-cell enrichment and gating, antibody clone and fluorochrome, instrument calibration, use of quantitative beads or standardized fluorescence intensity, sample handling, and a validated threshold. Marrow results may also fail to represent heterogeneous or extramedullary disease. Flow cytometry can therefore be incorporated as an exploratory, centrally harmonized biomarker in AISM research, but it should not currently be treated as a universally available or standalone clinical determinant (12, 21).

When a patient progresses after BCMA-directed therapy, AISM proposes a structured question rather than a fixed instruction: Is there sufficient evidence that an alternative target or mechanism should be prioritized? A GPRC5D-directed or FcRH5-directed approach may be reasonable when available and clinically appropriate, particularly after a short or absent response to BCMA-directed treatment. Reuse of BCMA-targeted treatment may still be appropriate in selected circumstances, particularly where the treatment modality differs, the interval is long, or alternative options are unavailable. The correct action should remain individualized and guided by trial eligibility, available evidence, and patient circumstances. **Figure 2** summarizes the candidate research constructs and their proposed components.

**Figure 2:**
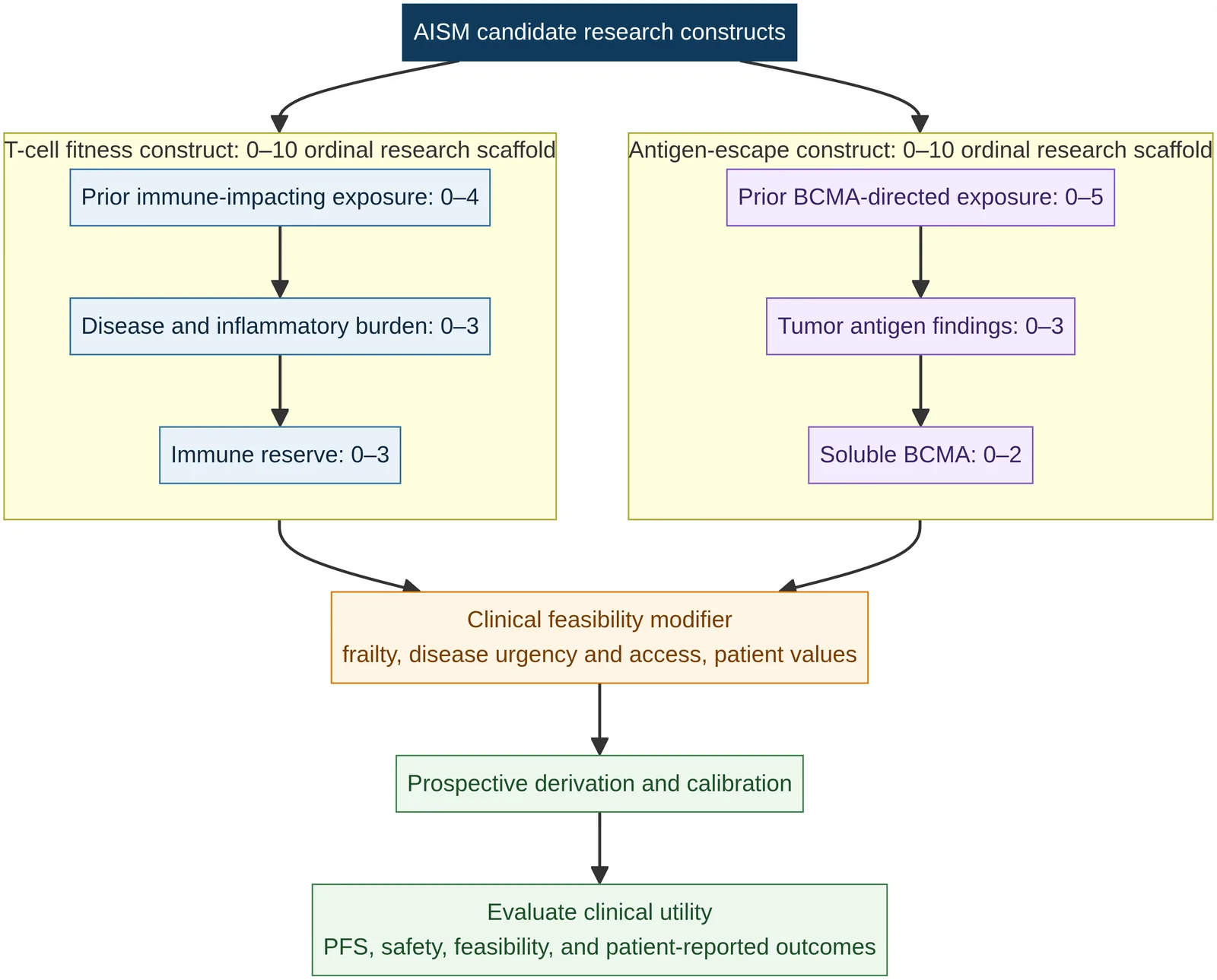
Candidate AISM research constructs. The provisional 0–10 T-cell fitness and antigen-escape constructs organize candidate measurements; clinical feasibility, disease urgency, and patient values modify interpretation. The scores are ordinal research scaffolds requiring prospective derivation, calibration, and validation, not clinical decision thresholds.

### 3.3 AISM clinical discussion matrix

**Table 3** presents the clinical question, relevant therapy discussion, evidence basis, and AISM-derived hypothesis. It is intended to support transparent multidisciplinary discussion alongside national and institutional guidance.

**Table 3:** AISM clinical discussion matrix.

Clinical context	AISM question or hypothesis	Potentially relevant therapy discussion	Evidence basis	AISM interpretation
Early relapse (1–3 prior lines), fit patient, CAR-T accessible within a clinically acceptable interval	Could earlier CAR-T referral preserve a favorable opportunity before additional immune-impacting therapy?	Discuss BCMA-directed CAR-T alongside approved alternatives; consider apheresis and bridging needs early.	Randomized clinical trial evidence: earlier-line CAR-T versus specified standard regimens (1, 2). Expert/consensus guidance: CAR-T before BCMA-directed BsAbs when feasible (6).	AISM conceptual hypothesis: earlier CAR-T referral may preserve a favorable treatment opportunity; superiority over every alternative sequence is unproven.
Rapidly progressive disease or high burden	Can the patient safely wait for apheresis, manufacturing, bridging, and CAR-T infusion?	Consider immediate, off-the-shelf therapy, including an appropriate BsAb such as linvoseltamab when clinically appropriate (22), while recognizing infection and toxicity management needs.	Prospective clinical evidence: single-arm BsAb studies (3–5). Expert/consensus guidance: clinical urgency and feasibility inform choice (6).	AISM conceptual hypothesis: off-the-shelf treatment may be prioritized when the CAR-T pathway is not clinically feasible.
Frailty, organ impairment, or barriers to CAR-T pathway	Does expected benefit of CAR-T outweigh lymphodepletion, hospitalization, travel, and caregiver burden?	Individualize between BsAb, CAR-T, other systemic therapy, or supportive approaches through shared decision-making.	Expert/consensus guidance and real-world feasibility considerations (6, 19, 23, 24).	Clinical feasibility modifier; no score-based allocation is proposed.
Progression after BCMA-directed immunotherapy	Is target change favored by prior response, interval, antigen assessment, and alternative resistance mechanisms?	Consider GPRC5D-directed therapy or investigational FcRH5-directed cevostamab (25), as well as clinical trials or selected BCMA reuse.	Retrospective/real-world evidence, biological/mechanistic evidence, and expert/consensus guidance (6, 11, 13, 14, 25, 26).	AISM conceptual hypothesis: target switching may be preferable in selected resistance contexts; same-target reuse remains possible in selected circumstances.
High-risk or extramedullary disease	Is spatial or clonal heterogeneity likely to limit single-target control?	Consider clinical trials of multi-target or combination approaches where appropriate.	Early-phase evidence and biological rationale; no established standard based solely on extramedullary disease.	Investigational setting; multi-target therapy should be studied rather than routinely prescribed.

This matrix separates the clinical question, potentially relevant therapy discussion, evidence basis, and AISM interpretation. It is a hypothesis-generating discussion aid, not a validated treatment-allocation algorithm.

## 4 Potential role of CAR-T as the first T-cell–redirecting therapy

### 4.1 Rationale for early CAR-T referral

Ciltacabtagene autoleucel and idecabtagene vicleucel have demonstrated major efficacy in RRMM, and phase 3 trials support movement of CAR-T into earlier relapse compared with specified standard regimens (1, 2). Trials of BsAb-based therapy in earlier lines, including peer-reviewed studies of teclistamab (27, 28) and preliminary conference-abstract data from the ongoing MagnetisMM-32 study (29), alongside the registered ongoing study (30), further illustrate the shift toward earlier T-cell redirection. These data establish earlier-line activity of several approaches; they do not by themselves establish a universal sequence in which CAR-T is superior to every BsAb-based strategy for every eligible patient.

AISM therefore advances a narrower hypothesis: in a patient with early RRMM who is eligible for CAR-T and can access a treatment center within a clinically acceptable interval, early referral, and where appropriate leukapheresis, may be worth prioritizing before prolonged exposure to a BCMA-directed BsAb. This hypothesis is consistent with emerging sequencing guidance that favors CAR-T before BCMA-directed BsAbs or antibody–drug conjugates when feasible (6). Its proposed mechanism is preservation of an option that depends on successful cell collection, manufacture, and *in vivo* expansion, rather than a claim that all patients have better outcomes with this sequence. Patient preference, toxicity history, clinical urgency, and alternative trials remain central.

A CAR-T–associated treatment-free interval can be meaningful to patients, but it is not synonymous with cure or superior health-related quality of life. Relapse remains common, and the interval can be complicated by prolonged cytopenias, recurrent infections, delayed neurologic events, secondary malignancies, and the logistical burden of monitoring. Any sequencing study should therefore report treatment-free time together with PFS, survival, safety, healthcare use, and patient-reported outcomes (1, 2, 19).

### 4.2 BsAbs as immediate therapy and possible post-target-switch salvage

BCMA-directed BsAbs such as teclistamab, elranatamab, and linvoseltamab, and the GPRC5D-directed BsAb talquetamab, offer off-the-shelf treatment with rapid initiation (3–5) (22). Their availability is particularly valuable for patients whose disease is progressing too quickly for the CAR-T pathway or for those who cannot undertake that pathway. AISM does not label BsAbs as second-best treatment; it identifies situations in which their immediacy, route of administration, and feasibility may be decisive.

After BCMA-directed treatment, target switching to a GPRC5D-directed or FcRH5-directed treatment provides a biologically coherent strategy when access and eligibility permit. Cevostamab, an FcRH5×CD3 bispecific antibody, remains investigational: phase 1 data support clinical activity, but they do not establish a preferred sequence or comparative benefit (25). However, sequencing should account for the entire resistance context rather than a presumed binary state of BCMA loss. A documented antigen alteration, a brief response to BCMA therapy, high sBCMA in a relevant assay, or relapse pattern may increase interest in a target switch. By contrast, longer interval since prior BCMA therapy, a change in modality, and limited alternatives may support discussion of BCMA re-treatment. Prospective studies are needed to identify patients in whom one strategy is more effective.

### 4.3 Real-world feasibility and implementation pathway

AISM would add value only if it can be implemented without creating an impractical layer of testing or worsening inequities. The CAR-T pathway requires referral networks, apheresis, manufacturing capacity, bridging strategies, inpatient and outpatient expertise, caregiver support, and reimbursement. BsAbs also require specialized step-up dosing, toxicity management, infection surveillance, access to immunoglobulin replacement, and frequent follow-up. Cost and availability differ substantially among health systems (23, 24). Consequently, a biologically attractive sequence may be infeasible or unsafe for an individual patient.

A pragmatic implementation approach should have two tiers. The minimum clinical tier would use information already available in routine practice: prior target exposure, disease kinetics, performance status and frailty, cytopenias, organ function, standard disease assessment, access to a cellular-therapy center, and patient preference. The research biomarker tier would add standardized lymphocyte phenotyping, representative tumor antigen assessment, sBCMA, and longitudinal immune and tumor sampling. The latter should be introduced through prospective protocols, not as a prerequisite that delays treatment. This approach would permit evaluation of whether additional biological information changes decisions, improves outcomes, or merely introduces complexity and resource demand.

Clinical trials often enroll patients with performance status, organ function, support, and center access that may not reflect routine practice. AISM validation must therefore include real-world populations, patients with frailty and comorbidities, geographic and economic barriers, and resource-limited settings. It should also test reproducibility across laboratories and treatment centers before any broad implementation is considered.

### 4.4 Multi-target approaches in high-risk disease

Extramedullary disease and other high-risk features can reflect tumor heterogeneity, aggressive biology, and a suppressive microenvironment. They may be reasons to consider a multi-target strategy or a clinical trial, but they are not sufficient evidence to prescribe dual targeting routinely. Early studies, including combination T-cell–redirecting approaches, are encouraging but require careful evaluation of efficacy, infectious risk, and cumulative immunosuppression. In AISM, multi-target therapy remains an investigational option rather than a default recommendation.

## 5 Measurable residual disease as a dynamic checkpoint

Minimal residual disease (MRD) negativity is associated with favorable outcomes in multiple myeloma and is a useful measure of depth of response (31). AISM treats MRD as a potential monitoring tool, not a validated trigger for escalation, de-escalation, or discontinuation of immunotherapy. The clinical meaning of sustained MRD negativity may differ by setting, treatment mechanism, assay sensitivity, sample quality, and duration of follow-up. Marrow-based MRD assessment can also miss spatially heterogeneous or extramedullary disease.

MRD conversion or sustained negativity can be incorporated into a prospective AISM study as an exploratory or key secondary endpoint. Whether intervening at MRD recurrence before clinical progression improves survival remains uncertain and is under investigation; the REMNANT study has so far been reported as an ASH abstract, so its findings should be regarded as preliminary (32). Likewise, the duration of MRD negativity needed to support treatment discontinuation is not established. AISM therefore does not advocate MRD-directed modification outside an appropriate protocol or established guidance.

## 6 Prospective validation agenda, limitations, and primary outcome

AISM should be tested as a sequence-level intervention, not merely correlated with biomarkers after treatment. The most clinically interpretable primary outcome for an initial comparative study would be PFS from randomized allocation to an AISM-informed sequencing strategy versus contemporary clinician-selected sequencing, with an event defined as disease progression or death. This endpoint directly tests whether the framework improves time without progression or death while avoiding an assumption that treatment-free time alone represents benefit.

Key secondary outcomes should include overall survival, response and duration of response, time to next treatment, duration of treatment-free interval, MRD status and durability, rates of severe infection and hypogammaglobulinemia, prolonged cytopenias, cytokine-release syndrome and neurotoxicity, healthcare utilization, feasibility of completing the planned sequence, cost and resource use, and patient-reported physical function and quality of life. Prespecified analyses should evaluate whether the model performs differently by prior BCMA exposure, frailty, disease burden, cytogenetic risk, extramedullary disease, and geographic access.

A staged research program is appropriate. First, a prospective observational cohort should collect harmonized baseline immune phenotyping, tumor antigen measures, sBCMA, treatment history, and outcomes to derive—not assume—the relative weight of candidate variables. Second, the resulting model should undergo independent external validation for calibration, discrimination, and clinical utility. Third, a pragmatic comparative trial should test AISM-informed sequencing against standard multidisciplinary decision-making. Embedded qualitative research should determine whether the framework improves shared decision-making or creates unwanted complexity.

Several limitations are immediate. The proposed scores are conceptual; no validated assay, weighting system, cutoff, or clinical action threshold currently exists. Surface antigen testing and sBCMA assays are not standardized across laboratories or treatment modalities. The biological mechanisms of resistance are multifactorial, and a two-domain model will necessarily be incomplete. Rapid evolution of trispecific antibodies, improved CAR-T products, and new target classes may change the sequencing landscape before the framework is fully evaluated. Most importantly, biological plausibility and associations from cross-trial or retrospective evidence cannot establish that a particular sequence improves outcomes.

## 7 Conclusion

The expanding range of T-cell–redirecting therapies in RRMM makes sequencing a central clinical challenge. Current recommendations increasingly recognize patient, disease, prior target exposure, access, and toxicity factors, yet a validated integrated framework is lacking. AISM is proposed as a transparent, hypothesis-generating model that brings host T-cell context, tumor target status, and practical feasibility into the same discussion.

At present, AISM is intended to structure research and multidisciplinary discussion. It offers testable propositions: early CAR-T referral may be advantageous for selected eligible patients when feasible; BsAbs may be essential when immediate therapy is required; and target switching after prior BCMA-directed therapy may be appropriate in some resistance contexts. Prospective, comparative, and real-world studies are needed to determine whether biologically informed sequencing based on AISM improves PFS or other outcomes compared with current approaches. Individualized multidisciplinary care and shared decision-making remain central.

## Data Availability

The raw data supporting the conclusions of this article will be made available by the authors, without undue reservation.
